# Comparison of ultrasonography learning between distance teaching and traditional methodology. An educational systematic review

**DOI:** 10.1590/1516-3180.2021.1047.R.19052022

**Published:** 2022-08-29

**Authors:** Márcio Luís Duarte, Lucas Ribeiro dos Santos, Wagner Iared, Maria Stella Peccin

**Affiliations:** I MD, MSc, PhD. Musculoskeletal Radiologist, WEBIMAGEM Telerradiologia, São Paulo (SP), Brazil; and Professor, Ultrasonography, Centro Universitário Lusíada (UNILUS), Santos (SP), Brazil.; Centro Universitário Lusíada, Santos, SP, Brazil; II MD, MSc. Endocrinologist and Professor, Physiology and Internal Medicine, Centro Universitário Lusíada (UNILUS), Santos (SP), Brazil; and Doctoral Student, Evidence-Based Health Program, Universidade Federal de São Paulo (UNIFESP), São Paulo (SP), Brazil.; Universidade Federal de São Paulo, Paulo, SP, Brazil; III MD, PhD. Supervisor Professor, Evidence-Based Health Postgraduate Program, Universidade Federal de São Paulo (UNIFESP), São Paulo (SP), Brazil.; IV PT, PhD. Associate Professor, Department of Human Movement Sciences and Advisor, Evidence-Based Health Postgraduate Program, Universidade Federal de São Paulo (UNIFESP), São Paulo (SP), Brazil.

**Keywords:** Ultrasonography, Telemedicine, Education, distance, Ultrasound, Distance learning, Web-based, M-learning, E-learning

## Abstract

**BACKGROUND:**

Use of the web for radiological education is an obvious application. Many computer-based teaching materials have been developed over recent years, and e-learning is becoming increasingly popular in medical schools.

**OBJECTIVE:**

To assess whether the effectiveness of distance-learning and/or e-learning, m-learning and web-based methods are equivalent to traditional methods.

**DESIGN AND SETTING:**

Systematic review of comparative studies of teaching techniques guided by Best Evidence Medical Education.

**METHODS:**

A search was carried out in the MEDLINE, EMBASE, Cochrane Library, Tripdatabase, CINAHL and LILACS online databases in April 2020, for original publications in all languages. The following MeSH terms were used: Ultrasonography; Teleradiology; Telemedicine; Education, Medical; Teaching; and Simulation Training; along with the terms e-learning, m-learning and web-based. All eligible studies were assessed using the Kirkpatrick model and Buckley's quality indicators.

**RESULTS:**

The search in the databases and a manual search resulted in 4549 articles, of which 16 had sufficient methodological quality for their inclusion. From analysis of these data, it was observed that teaching of ultrasonography using telemedicine methods is similar to the traditional method, except for venous access procedures, for which the studies did not show agreement.

**CONCLUSION:**

We found that learning via telemedicine methodologies presents great acceptance among students, besides demonstrating quality similar to the traditional method. Thus, at least at the moment, this has the capacity to serve as an important adjunct in the teaching of ultrasonography.

**REGISTRATION NUMBER:**

DOI: 10.17605/OSF.IO/CGUPA at the OPENSCIENCE Framework.

## INTRODUCTION

Learning is an event consisting of a goal, a training activity and an appraisal.^1^ The aim is to have a total instructional experience, associated with the usual descriptors.^1^ The method for acquiring further information is not merely a matter of obtaining data (surface learning); additionally, it involves the capacity to interpret it and feasibly do this.^2^

One essential feature of the learning method relates to student motivation.^3^ Student motivation involves mutual communications within ambient circumstances, actions and particular aspects of these.^3^ This automated manner of learning develops when learners become self-aware administrators of their own motivation and performance, in order to reach the desired goals.^3^ Fun is also a meaningful part of learning events and, perhaps, can be one of the principal components, with self-determination, towards achievement of problem-based learning within health-related teaching.^4^

Undergraduate, postgraduate and continuing professional development studies compose medical education.^2^ All trainees have their limitations, skills and decision-making capacity.^2^ The job of mentors is to provide an atmosphere and resources within which any trainee can develop.^2^

There is a lack of formal teaching time in medical schools dedicated to interpretation of radiological images.^5^ This situation is disappointing, given that imaging can be used as a dynamic teaching utility, to demonstrate anatomy, pathology and physiology.^5^ Medical students develop the way they learn, but their progression does not always go from duality to multiplicity.^2^

Health-related teaching needs a diversity of elements, comprising institutional, visual, concrete and accurate knowledge.^6^ Conventional health-related teaching includes use of books, speeches, pictures and guidelines.^6^ The value of lectures within teaching has been challenged historically, and investigations have revealed that they have insufficient influence on short and, notably, long-term retention, particularly with regard to expositions that last for longer than 20 minutes.^7^ Just 20% to 30% of the information imparted in any given session can be put into practice by trainees immediately following the exposition. Moreover, over the subsequent two weeks, 90% of the data is wasted.^7^

Low-cost telemedicine technologies can enable doctors to access expert support, remote procedure guidance and real-time training opportunities, thereby reducing unnecessary transportation costs and improving patient outcomes.^8–11^ There are studies in the medical literature that have reported that doctors who were trained remotely over the internet had a good degree of satisfaction with the quality of their training and achieved a quality level in evaluating ultrasound images that was similar to that of doctors who underwent in-person training.^5,12^

## OBJECTIVE

The objective of this systematic review was to assess whether the effectiveness of distance-learning and/or e-learning, m-learning and web-based methods, for ultrasonography training, is equivalent to traditional methods.

## METHODS

### Study model

The reference point for this study was the education-oriented systematic review model for Best Evidence Medical Education (https://www.bemecollaboration.org/). The study was registered on the OpenScience Framework platform (https://osf.io/wn762). This study was considered exempt from formal institutional review by our institutional review board because no human or animal subjects were studied.

### Modalities of distance-learning

- Electronic learning (E-learning) is an online educational assistance website that instructs and enables students to enhance specific topics.
- Mobile learning (m-Learning) can be described generically as a modality of e-Learning in which learning takes place through easy-to-handle mobile electronic devices (such as smartphones and tablets, for example).
- A video lesson is a video that presents educational material to a subject.
- A live distance class is an online class at a regularly planned time in which learners work together with their instructor and classmates at the same time on the same days. Homework tasks are then accomplished outside of this dedicated lesson period, just like in in-person lessons.

### Search strategy

A systematic search of the literature was carried out on April 16, 2020, in the following online databases: Medline (PubMed); EMBASE; Cochrane Library; LILACS; Tripdatabase; CINAHL; ERIC; and SciELO. Original published articles in any language were sought using the following MeSH terms: Ultrasonography; Distance-learning; Online learning; Teleradiology, Telemedicine; Education, Medical; Medical Education Online; Simulation Training; and Teaching. In addition, the terms e-learning, m-learning and web-based were also used. The reference lists of studies that were included and those of the main reviews on this subject were also evaluated. Manual searches were also carried out in the reference lists. All of these searches are shown in Table 1.

**Table 1:** Search strategy according to the corresponding database

Database	Search strategy
Cochrane Library	#1: MeSH descriptor: [Ultrasonography] explode all trees #2: MeSH descriptor: [Education, Distance] explode all trees #3: MeSH descriptor: [Teleradiology] explode all trees #4: MeSH descriptor: [Telemedicine] explode all trees #5: MeSH descriptor: [Education, Medical] explode all trees: #6: MeSH descriptor: [Simulation Training] explode all trees #7: MeSH descriptor: [Teaching] explode all tress #8: #1 AND #2 OR #3 OR #4 AND #5 OR #6 OR #7
MEDLINE	#1: “Ultrasonography”[Mesh] OR (Echotomography) OR (Diagnostic Ultrasound) OR (Diagnostic Ultrasounds) OR (Ultrasound, Diagnostic) OR (Ultrasounds, Diagnostic) OR (Sonography, Medical) OR (Medical Sonography) OR (Ultrasound Imaging) OR (Imaging, Ultrasound) OR (Imagings, Ultrasound) OR (Ultrasound Imagings) OR (Echography) OR (Ultrasonic Imaging) OR (Imaging, Ultrasonic) OR (Echotomography, Computer) OR (Computer Echotomography) OR (Tomography, Ultrasonic) OR (Ultrasonic Tomography) OR (Diagnosis, Ultrasonic) OR (Diagnoses, Ultrasonic) OR (Ultrasonic Diagnoses) OR (Ultrasonic Diagnosis) #2: “Teleradiology”[MeSH] OR “Telemedicine”[MeSH] OR (mobile health) OR (health, mobile) OR (health) OR (telehealth) OR (ehealth) OR “m-learning” OR “e-learning” OR “web based” OR “Education, Distance”[MeSH] OR (distance education) OR (distance learning) OR (learning, distance) OR (online learning) OR (learning, online) OR (online education) OR (education, online) OR (online education) OR (correspondence courses) OR (correspondence course) OR (course, correspondence) #3: “Education, Medical”[MeSH] OR (medical education) OR “Simulation Training”[MeSH] OR (training, simulation) OR (interactive learning) OR (learning, interactive) OR “Teaching”[MeSH] OR (training techniques) OR (technique, training) OR (techniques, training) OR (training technique) OR (training technics) OR (technic, training) OR (technics, training) OR (training technic) OR (pedagogy) OR (pedagogies) OR (teaching methods) OR (method, teaching) OR (methods, teaching) OR (teaching method) OR (academic training) OR (training, academic) OR (training activities) OR (activities, training) OR (training activity) OR (techniques, educational) OR (technics, educational) OR (educational technics) OR (educational technic) OR (technic, educational) OR (educational techniques) OR (educational technique) OR (technique, educational) #4: #1 AND #2 AND #3
EMBASE	#1: Echography/exp #2: Online learning/exp OR online education/exp OR teleradiology/exp OR telemedicine/exp OR e learning OR m-learning #3: Medical education/exp OR medical education training/exp OR Simulation training/exp OR Clinical education/exp OR Teaching/exp #1 AND # 2 AND #3
LILACS	#1: mh:”Ultrassonografia”/exp OR (Ultrasonografía) OR (Ultrasonography) OR (Ecografia) OR (Ecotomografia Computador) OR (Sonografia Médica) OR (Ecografia Médica) OR (Tomografia Ultrassônica) OR (Diagnóstico Ultrassom) OR (Imagem Ultrassônica) OR (Imagem Ultrassonográfica) OR (Imagem Ultrassom) OR (Imagem Ultrassom) OR (Ecotomografia) OR (mh:E01.370.350.850$) #2: mh:” Educação a Distância”/exp OR (Educación a Distancia) OR (Education, Distance) (Correspondence Course) OR (Correspondence Courses) OR (Course, Correspondence) OR (Cyberlearning) OR (Distance Education) OR (Distance Learning) OR (Education, Online) OR (Interactive Tele-Education) OR (Learning, Distance) OR (Learning, Online) OR (Online Education) OR (Online Educations) OR (Online Learning) OR (Tele-Education) OR (Teletraining) OR (eLearning) or(mh:I02.195$) OR (mh:SP2.021.167.010.090.030$) OR (mh:SP2.021.172.010.099$) OR (mh:SP2.031.332.030$) OR (mh:SP4.017.047.599$) OR (SP4.127.428.764) OR mh:”Teleradiology”/exp OR (Telerradiología) OR (Telerradiologia) OR (mh:E05.920.700$) OR (mh:H02.010.850.700$) OR (mh:H02.403.840.700$) OR (mh:L01.178.847.652.700$) OR (mh:N04.452.515.825.500$) OR (mh:N04.590.374.800.700$) OR (mh:SP2.021.167.010.090.210$) OR (mh:SP2.031.332.210$) OR (Telemedicine) OR (Telemedicina) OR (mh:H02.403.840$) OR (mh:L01.178.847.652$) OR (mh:N04.590.374.800$) OR (mh:SP2.016.303$) OR (mh:SP2.021.167.010.090$) OR (mh:SP2.031.332$) #3: mh: “Education, Medical”/exp OR (Educación Médica) OR (Educação Médica) OR (Medical Education) OR (mh: I02.358.399$) OR #4: mh: “Simulation Training”/exp OR (Entrenamiento Simulado) OR (Treinamento por Simulação) OR (Interactive Learning) OR (Interactive Learning) OR (Training, Simulation) OR (mh: I02.903.847$) OR mh: “Ensino”/exp OR (Enseñanza) OR (Teaching) OR (Academic Training) OR (Activities, Training) OR (Educational Technic) OR (Educational Technics) OR (Educational Technique) OR (Educational Techniques) OR (Method, Teaching) OR (Methods, Teaching) OR (Pedagogies) OR (Pedagogy) OR (Teaching Method) OR (Teaching Methods) OR (Technic, Educational) OR (Technic, Training) OR (Technics, Educational) OR (Technics, Training) OR (Technique, Educational) OR (Technique, Training) OR (Techniques, Educational) OR (Techniques, Training) OR (Training Activities) OR (Training Activity) OR (Training Technic) OR (Training Technics) OR (Training Technique) OR (Training Techniques) OR (Training, Academic) OR (mh:I02.903$) #4: #1 AND #2 AND #3
Tripdatabase	(title:Ultrasonography)(title:Teleradiology OR Telemedicine OR Education, Distance OR e-learning OR m-learning OR Online learning)(Education, Medical OR Medical Education Training OR Simulation Training OR Teaching)
CINAHL	#1: (Ultrasonography) #2: (Telemedicine & e-Health) OR (Distance Education) OR (Medical Education Online) #3: (Medical Education) #4: #1 and #2 and #3
ERIC	#1: MeSH descriptor: Ultrasonography #2: MeSH descriptor: Education, Distance #3: MeSH descriptor: Teleradiology #4: MeSH descriptor: Telemedicine #5: MeSH descriptor: Education, Medical #6: MeSH descriptor: Simulation Training #7: MeSH descriptor: Teaching #8: #1 AND #2 OR #3 OR #4 AND #5 OR #6 OR #7
SciELO	#1: MeSH descriptor: Ultrasonography #2: MeSH descriptor: Education, Distance #3: MeSH descriptor: Teleradiology #4: MeSH descriptor: Telemedicine #5: MeSH descriptor: Education, Medical #6: MeSH descriptor: Simulation Training #7: MeSH descriptor: Teaching #8: #1 AND #2 OR #3 OR #4 AND #5 OR #6 OR #7

The search was performed in accordance with the Preferred Reporting Items for Systematic Reviews and Meta-Analyses (PRISMA) guidelines. Studies that compared the teaching of ultrasonography for healthcare professionals, between traditional methods and electronic means through distance-learning, e-learning, m-learning and web-based learning, were included regardless of their publication status.

There was no language restriction. There was no exclusion for population size or age. There was no funding for this study. The PICO technique (Population, Intervention, Comparison, Outcome) was used to define the question and the development of the research, as follows:

P = Undergraduate health care students; postgraduate trainees; continuous professional development training – independent of the specialties.

I = Distance-learning to teach ultrasonography.

C = Traditional methodology versus distance-learning.

O = Improved ultrasound skills, to achieve an accurate diagnosis

### Selection of studies and data extraction

The study selection process was carried out by two independent reviewers and any disagreement was resolved by a third reviewer. The selection of studies was carried out in two stages. In the first stage, the titles and abstracts of the references identified through the search strategy were evaluated and the potentially eligible studies were preselected. In the second stage, a full-text evaluation of the preselected studies was carried out to confirm their eligibility. In cases of disagreement, a third author was consulted. Data extraction was performed using a standardized form. The outcomes analyzed were the score previously established for the training method and the performance of the procedure.

The selection process was carried out through the Rayyan platform (https://rayyan.qcri.org).13

### Quality assessment

All eligible studies were assessed using Buckley's quality indicators^14^ and the Kirkpatrick training assessment model described in BEME Guide No. 8 by Steinert et al.^15^ These tools are based on instruments that cover a wide range of methodological issues in studies on evaluation of teaching methodologies.

## RESULTS

**Figure 1:**
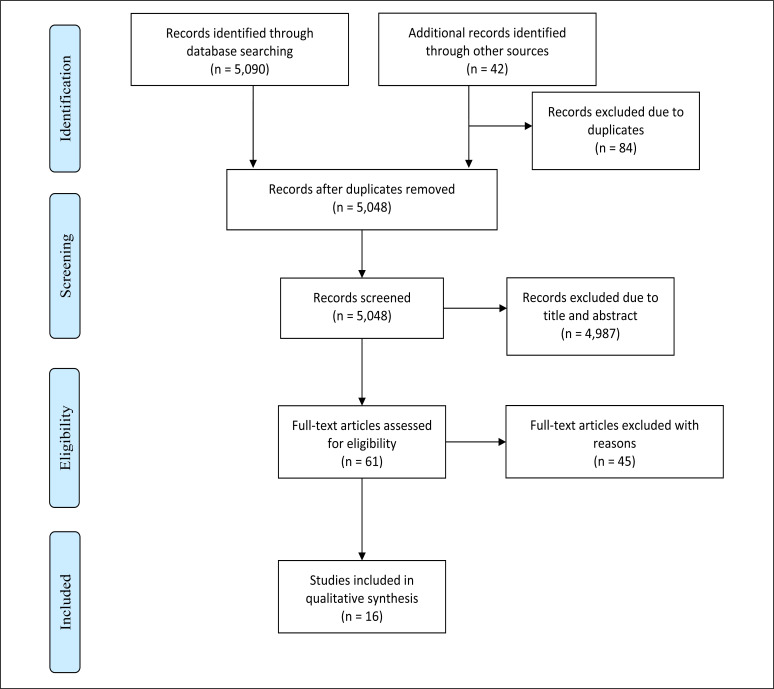
PRISMA flow diagram.

The search in the databases yielded 5,090 articles. Additionally, seven articles were found through a manual search. After excluding duplicates, 5,048 articles were screened, out of which 61 were evaluated in their entirety; from these, 16 presented sufficient methodological quality for their inclusion (Figure 1). The study by Socransky et al.^16^ was excluded due to loss to follow-up of over 50% of the initial participants. Hempel et al.^17^ was not included because its results from the study phase that were compatible with our inclusion criteria had already been published previously.^7^

Eleven studies evaluated teaching among doctors and/or residents and/or medical school students;^7,18–27^ one study conducted in England evaluated teaching among nurses,^28^ and four studies carried out in Spain evaluated physical therapy students.^4,29–31^

Regarding methodology, one study was a cross-sectional, randomized study,^28^ one was a prospective pseudorandomized study,^25^ one was a prospective cohort^19^ and three were randomized controlled trials.^4,7,18,20–24,26,27,29–31^

In ten studies, a questionnaire was administered both before and after each teaching technique was applied.^7,18–21,23–25,27–29^ In three of these,^19,20,27^ a questionnaire was also administered long after the last lesson, as a late assessment of knowledge retention. In three studies, no questionnaire was used;^22,26,30^ while in two studies a questionnaire was administered only after the teaching technique.^4,31^ In six studies, teaching of the FAST ultrasound technique (directed ultrasound in trauma cases),^24,25^ or structures included in this,^28^ was evaluated; or point-of-care was evaluated.^7,21,27^

One study assessed thoracic structures,^19^ and one was specifically directed to pneumothorax.^20^ Three studies evaluated interventions: venous access,^18^ arterial access^22^ and intravenous central catheter.^26^ Five studies evaluated the teaching of structures of the musculoskeletal system.^4,23,29–31^ Five studies did not involve any practical evaluation, and their results were based only on questionnaires carried out after applying the teaching technique.^18,19,23–25^

Arroyo-Morales et al.^31^ conducted a randomized clinical trial to evaluate the learning of knee ultrasound therapy among 44 students who were divided into two groups: traditional method and textbooks associated with e-learning. At the end of the study, they reported that the two groups obtained similar results in the theoretical evaluation; however, in the practical evaluation with ultrasound, the students in the e-learning group obtained higher-quality images despite taking longer to perform the examination. It should be noted that the students in the e-learning group showed good acceptance of distance-learning.

Bertran et al.^26^ also conducted a randomized clinical trial, in which they evaluated 43 residents in anesthesiology regarding positioning of the central venous catheter guided by ultrasound. They concluded that the residents who had had a video lesson showed better results than those who had had a classroom lesson.

Ten nurses participated in a randomized comparative cross-sectional study by Brisson et al.^28^ in which they were learning about the Morrison space. The subjects were divided into two groups: telemedicine and face-to-face group (classroom lesson). At the end of the study, teaching by means of telemedicine proved to be equivalent to classroom lessons for acquiring the practical skill of ultrasound, and this was achieved within similar times.

In a randomized clinical trial by Cantarero-Villanueva,^30^ teaching of lumbopelvic ultrasonography was evaluated among 44 physiotherapy students. It was concluded that the e-learning group showed better results than the control group, which used books, and that this could be an effective adjunctive strategy for teaching.

Chenkin et al.^18^ conducted a randomized clinical trial in which only theoretical ultrasonography was assessed, with no evaluation of ultrasound practice, among 21 emergency department doctors and residents. They found that the group that received an internet-based tutorial was at least as effective as the group who had attended an in-person teaching lecture on ultrasound-guided venous access.

Furthermore, in the prospective cohort study by Cuca et al.,^19^ no assessment of ultrasonography practice was conducted. There were only assessments via questionnaires regarding thoracic structures in ultrasonography, including two post-tests. That study evaluated 75 doctors and medical students and it was found that teaching via e-learning showed results that were similar to those of the traditional method, including through a survey conducted two weeks after the teaching, which evaluated the retention of information.

In a randomized clinical trial by Edrich et al.,^20^ 138 anesthesiologists were assessed. They were divided into three groups: a group without instruction, a group with classroom instruction and a group that received instruction through telemedicine. It was concluded that teaching via telemedicine provided results that were similar to those through the traditional methodology, including in a questionnaire administered four weeks after the teaching, to evaluate the retention of information.

Fernández-Lao et al.^29^ carried out a randomized clinical trial among 49 physiotherapy students. They concluded that the group with m-learning showed better positioning and handling of the transducer, and patient positioning, than the group with traditional methodology for shoulder ultrasound assessment.

Haskins et al.^21^ evaluated 18 anesthesiology residents through a randomized clinical trial. They found that there was no evidence of difference between the traditional teaching and e-learning groups regarding the learning results or satisfaction, in relation to point-of-care ultrasound. Hempel et al.^7^ analyzed 60 medical students from the third year of an undergraduate course in a randomized clinical trial and, like Haskins et al.,^21^ found that teaching via e-learning showed results similar to those of the traditional method, regarding point-of-care ultrasonography.

Lian et al.^22^ evaluated 30 medical students in three groups: traditional method, e-learning and no previous instruction. Through this randomized clinical trial, they analyzed the teaching of ultra-sound-guided vascular access and concluded that the traditional method group achieved significantly better performance than the e-learning group and the uneducated group.

Lozano-Lozano et al. conducted a randomized clinical trial^4^ in which the teaching of 105 physiotherapy students for evaluating sports pathological conditions using ultrasound was evaluated. They concluded that the m-learning group achieved better patient positioning, transducer management and image adjustment than the traditional method group. However, less time was required for performing the examination through the traditional method.

In a randomized clinical trial by Maloney et al.,^23^ theoretical teaching of musculoskeletal ultrasonography was evaluated among 33 radiology residents, without any practical evaluation. It was concluded that the group with the traditional teaching methodology presented a result that was slightly better than that of the e-learning group (less than 5% difference).

Platz et al. carried out two studies analyzing FAST, but without evaluation of ultrasound practice. One was a prospective pseudorandomized trial^25^ among 55 doctors and residents of different specialties divided into three groups: traditional method, tele-medicine method and no previous instruction. From this, it was concluded that telemedicine teaching presented results similar to those of the traditional method. The other was a randomized clinical trial^24^ among 44 emergency and surgery residents, in which it was found that computer-based classes were not inferior to classroom classes among individuals without previous training, for teaching about FAST ultrasound.

Soon et al.^27^ carried out a randomized clinical trial on point-of-care ultrasound for pleural effusion and pneumothorax, among 45 pediatric physicians without experience of ultrasound. These subjects were divided into two groups: web classroom and in-person classroom, followed by practice on living models. They concluded that teaching via the web was at least as effective as the usual teaching method, including with regard to evaluation of information retention, among 39 of the study participants, conducted two months later.

A summary of all the studies is presented in Table 2.^4,7,18–31^

**Table 2:** Summary of study findings

Study/year	Country	Design	Students	Examination	Intervention	Comparator	Results	Kirkpatrick
Arroyo-Morales et al.^31^/2012	Spain	Randomized clinical trial with questionnaire only after intervention	44 second-year physiotherapy students	Knee	E-learning - ECOFISIO + books and texts	Books and texts	No difference in the theoretical part. Practice: e-learning group took longer, but the image was better. E-learning was better in the questionnaire. The students liked the site.	3
Bertran et al.^26^/2017	France	Randomized clinical trial without questionnaire	43 residents in anesthesiology and intensive care	Positioning of the central venous catheter	Video lesson	Classroom lesson	Residents who had a video lesson showed better results than those who had an in-person class.	2B
Brisson et al.^28^/2015	Canada	Comparative cross-sectional randomized study with questionnaire pre and post-intervention	10 nurses	Morrison space evaluation	Live distance class	Classroom lesson	Telemedicine teaching is equivalent to classroom lesson for the acquisition of practical ultrasound skills and in a similar time.	3
Cantarero-Villanueva et al.^30^/2012	Spain	Randomized clinical trial without questionnaire	44 physiotherapy students	Lumbopelvic region	E-learning - ECOFISIO + books and texts	Books and texts	E-learning can be an effective adjunct strategy in teaching.	3
Chenkin et al.^18^/2008	Canada	Randomized clinical trial with questionnaire before and after intervention	21 participants between staff physicians and residents of the emergency sector	Positioning of the central venous catheter	E-learning	Books and texts	Web-based tutorial was at least as effective as a teaching lecture. Did not carry out a practical evaluation.	2B
Cuca et al.^19^/2013	Germany	Prospective cohort study with a questionnaire before and after intervention, plus a post-intervention sustainability questionnaire two weeks later	75 doctors and medical students	Chest and lungs	E-learning	Classroom lesson	Teaching by e-learning showed results similar to the traditional method. Did not carry out a practical evaluation.	2B
Edrich et al.^20^/2016	United States; Germany; Austria	Randomized clinical trial with a questionnaire before and after intervention, plus a post-intervention retention questionnaire four weeks later	138 anesthesiologists	Chest and lungs	E-learning	Classroom lesson; No teaching technique	Telemedicine teaching showed results similar to the traditional method.	3
Fernández-Lao et al.^29^/2016	Spain	Randomized clinical trial with questionnaire before and after intervention	49 physiotherapy students	Shoulder	M-learning - ECOFISIO + books and texts	Books and texts	The group with m-learning showed better positioning of the patient and transducer and better handling of the probe.	3
Haskins et al.^21^/2018	United States	Randomized clinical trial with questionnaire before and after intervention	18 anesthesiology residents	Point-of-care	E-learning	Classroom lesson	There was no evidence of a difference between traditional teaching groups and e-learning in learning or satisfaction results.	3
Hempel et al.^7^/2016	Germany	Randomized clinical trial with questionnaire before and after intervention	60 third-year medical students	Point-of-care	E-learning	Classroom lesson	Teaching by e-learning showed results similar to the traditional method.	3
Lian et al.^22^/2017	Australia	Randomized clinical trial without questionnaire	30 medical students	Positioning of central venous catheter	E-learning	Classroom lesson; No teaching technique	The traditional method group performed significantly better on ultrasound-guided vascular access than those who did not receive training and e-learning.	2B
Lozano-Lozano et al.^4^2020	Spain	Randomized clinical trial with questionnaire only after intervention	105 physiotherapy students	Sports pathologies	M-learning - ECOFISIO + books and texts	Books and texts	The m-learning group had better patient positioning, transducer handling and image adjustment, but took more time than the control group. The students found it very useful.	3
Maloney et al.^23^/2016	United States	Randomized clinical trial with questionnaire before and after intervention	33 radiology residents	Musculoskeletal	E-learning	Classroom lesson	The difference in quality from traditional to e-learning was small (less than 5%). Did not carry out a practical evaluation.	2B
Platz et al.^25^/2010	Germany	Prospective pseudorandomized study with pre and post intervention questionnaire	64 doctors and residents: anesthesiologists, surgeons, internal medicine specialists and orthopedists	FAST	E-learning	Classroom lesson; No teaching technique	Telemedicine teaching showed results similar to the traditional method. Did not carry out a practical evaluation.	2B
Platz et al.^24^/2011	Germany	Randomized clinical trial with questionnaire before and after intervention	44 emergency and surgery residents	FAST	E-learning	Classroom lesson	Classes on computer are not inferior to school attendance regarding FAST ultrasound. Did not carry out a practical evaluation.	2B
Soon et al.^27^/2020	United States	Randomized clinical trial with a questionnaire before and after intervention, plus a post-intervention retention questionnaire two weeks later	45 pediatric physicians	Point-of-care	E-learning	Classroom lesson	Web-based learning is at least as effective as the usual classroom.	2B

FAST = focused assessment with sonography in trauma.

## DISCUSSION

Out of the 16 studies analyzed, nine^7,18–21,24,25,27,28^ showed similar results between the traditional and telemedicine groups. It should be noted that in four of these studies^18,19,24,25^ there was no practical evaluation; in these, assessments were only made through questionnaires that were administered before and after the teaching intervention.

In five studies,^4,26,29–31^ it was demonstrated that distance-learning was superior. Moreover, among these five studies, four^4,29–31^ evaluated physical therapy students; in two of these studies, m-learning technology was used,^4,29^ while e-learning technology was used in the other two.^30,31^ Two studies^22,23^ showed that the traditional education group had slightly better results than the telemedicine group that used e-learning technology. We need to contextualize that all these studies were carried out before the COVID-19 pandemic. Although the study by Lozano-Lozano et al.^4^ was published in 2020, it was carried out in 2014-2015.

In the present-day world, people acquire information daily through computers and, especially, smartphones. The current generation of students uses electronic media regularly, such that this is an essential part of their daily lives and modifies their brain structures in relation to learning. Thus, 37% of healthcare students have already used an application to develop their professional skills.^32^ Therefore, there is a need to adapt teaching methods. In this regard, the use of traditional teaching tools is now out of context.^4^

A study by Gul et al. showed that medical students prefer tele-medicine teaching over classroom lesson approaches, with regard to the clarity of procedures, the ability to ask questions and the quality of time spent learning, even in relation to surgical procedures.^28,33^ The most evident advantage of telemedicine teaching was the better positioning of the patient and handling of the transducer.^4,29^ Weber et al.^34^ also reported that there was a marked improvement in performance in the early stages of teaching; however, in their study, only the telemedicine group used augmented reality together with telementoring, which could be characterized as a form of bias.

Regarding studies on ultrasound-guided procedures, there was no agreement among the results. Bertran et al.^26^ compared teaching of central venous access by video and by the traditional method and concluded that residents who took video classes obtained better results. Lian et al.^22^ carried out a similar study comparing the teaching of vascular access by e-learning and the traditional method; they found that the traditional method showed significantly better results. In a randomized clinical trial by Chenkin et al.,^18^ a web tutorial teaching venous access was compared with an in-person lecture and it was concluded that the methods were equivalent.

Three studies^4,30,31^ from the University of Granada compared e-learning using a cell phone app versus books and texts, and another study^29^ compared m-learning versus books and texts. It was concluded from these four studies that the app was at least as effective as a teaching lecture, but that sometimes more time was needed for performing the ultrasound examination. Chenkin et al.^18^ reached the same outcome when teaching venous access in the University of Toronto. It needs to be noted that cell phones or tablets do not have present certain issues that relate to books, such as their weight (cell phones and tablets can store many books, independent of their volume and cost). It is also important to remember, on the other hand, that reading books on an electronic device is not always a pleasant experience for the learner and, thus, some students prefer textbooks on paper.

From gathering this data together, we can infer that ultra-sound telemedicine teaching methods are similar to the traditional method, except in the case of teaching venous access procedures, for which the studies did not show agreement. The studies demonstrated that telemedicine teaching was effective in relation to teaching thoracic ultrasound, FAST ultrasound, point-of-care and musculoskeletal ultrasound. Hempel et al.^17^ reported that use of social networks after the e-learning course presented superior results only when compared with classroom lessons. Student satisfaction compared between teaching methods was also similar, according to the studies evaluated.

The benefits of learning from computers are numerous and include interactivity, novelty, flexible programming, teachers’ relief from the need to give repetitive lectures and greater consistency in quality.^16,35,36^ The disadvantages of computer-based instruction include the lack of human interaction and guidance, the material presented in a format that is less pleasant to read than in a textbook and the possibility for a student to have unanswered questions.^7,23,35^

Use of the web for radiological education is an obvious application.^37–39^ Many computer-based teaching materials have been developed over recent years. M-learning, which is defined as “the ability to access educational resources, tools and materials anywhere, using a mobile device (smartphone)”,^4,29,40^ along with e-learning, is becoming increasingly popular in medical schools. Guides on the implementation of e-learning have been appearing.^6,18,22,30,31,35,41–44^ This method of learning has many organizational advantages over classroom lessons, as follows:^5,7,17,19,20,25,27,30,31,41^

- Environment free from stressful factors and without judgment.
- Live updates.
- Easy and uniform dissemination of teaching resources for teachers.
- Temporal and spatial flexibility for students.
- Greater accessibility.

The monetary savings that accrue through use of these new teaching methods should also be taken into account. Professionals in rural areas need to travel to major centers to receive medical education and training;^28,45^ alternatively, trained professionals from the main centers need to travel to teach in remote areas.^8,28,46^ Both of these situations are time-consuming and expensive.^8,28,46^ Telemedicine education offers an economical alternative for teaching skills in remote environments or for situations in which resources are limited.^8,28,45,47^

One limitation of this systematic review was that it seemed that many types of ultrasound examinations have not been evaluated in primary studies on distance-learning techniques in the medical literature, such as examinations on the thyroid, neck, breasts and prostate. In addition, because of the variability of outcomes between studies, performing a meta-analysis was not possible.

Regarding the implications for research, distance-learning techniques can be expanded to other areas of healthcare, such as biomedicine and nutrition, among others. Evaluations among medical specialties that remain little explored also need to be undertaken. It should be noted that none of the studies presented level 4 of the Kirkpatrick model (changes in system/organizational practice and changes among participants, students, residents or colleagues). Studies at level 4 could confirm the good results that were shown by the studies at levels 2B and 3 that were found. Another possibility that needs to be better explored is to combine these technologies with augmented reality and virtual reality, which would facilitate teaching in relation to areas that are difficult to access. Such combinations have already been successfully demonstrated with regard to obstetric examinations, in a study published by Zimmermann et al.,^48^ and this should be extended to other ultra-sound examinations.

Therefore, we can state that teaching of ultrasound by means of telemedicine is a novelty that is being implemented in the 21^st^ century. It presents possibilities such as videos and texts on computers or cell phones, with use of the internet and applications and/or programs, in addition to the possibility of augmented reality, which has already been analyzed in some studies. Thus, a new teaching technique is presented here, which is available to teachers for implementation, with the possibility of recording classes and making them available for repeated student viewing. This is something that is often impossible with classroom lessons. It should be noted that not all the studies evaluated here included practical analyses on ultrasound. However, with regard to the theoretical part of teaching, distance-learning presents results similar to traditional methods in the classroom.

## CONCLUSION

In this systematic review, we found that learning by means of telemedicine methodologies is widely accepted by students. Distance-learning can have quality similar to the traditional method and, at least at present, it can serve as an important adjunct in the teaching of ultrasonography, especially in relation to places that are difficult to access, where there are no schools/universities where this teaching could take place.

However, instructors need to pay attention to each student's particularities. Some students might not adapt to or appreciate the techniques of online teaching because of low levels of interaction between people or the need to study using a textbook rather than a screen. The need for internet access in order to have live video classes may be a problem for some locations. Studies conducted using this technology during the COVID-19 pandemic will provide new data on this technology. In addition, studies are still needed to assess the practical part of teaching ultrasonography at a distance.

## References

[1] Silva AB, de Amorim AC (2009). A Brazilian educational experiment: teleradiology on web TV. J Telemed Telecare.

[2] Taylor DC, Hamdy H (2013). Adult learning theories: implications for learning and teaching in medical education: AMEE Guide No 83. Med Teach.

[3] Ferrer-Torregrosa J, Torralba J, Jimenez MA, García S, Barcia JM (2015). ARBOOK: development and assessment of a tool based on augmented reality for anatomy. J Sci Educ Technol.

[4] Lozano-Lozano M, Galiano-Castillo N, Fernández-Lao C (2020). The Ecofisio Mobile App for Assessment and Diagnosis Using Ultrasound Imaging for Undergraduate Health Science Students: Multicenter Randomized Controlled Trial. J Med Internet Res.

[5] Salajegheh A, Jahangiri A, Dolan-Evans E, Pakneshan S (2016). A combination of traditional learning and e-learning can be more effective on radiological interpretation skills in medical students: a pre- and post-intervention study. BMC Med Educ.

[6] Zafar S, Safdar S, Zafar AN (2014). Evaluation of use of e-Learning in undergraduate radiology education: a review. Eur J Radiol.

[7] Hempel D, Sinnathurai S, Haunhorst S (2016). Influence of case-based e-learning on students’ performance in point-of-care ultrasound courses: a randomized trial. Eur J Emerg Med.

[8] Smith A, Addison R, Rogers P (2018). Remote Mentoring of Point-of-Care Ultrasound Skills to Inexperienced Operators Using Multiple Telemedicine Platforms: Is a Cell Phone Good Enough?. J Ultrasound Med.

[9] Kolbe N, Killu K, Coba V (2015). Point of care ultrasound (POCUS) telemedicine project in rural Nicaragua and its impact on patient management. J Ultrasound.

[10] Borbás J, Forczek E, Sepp R, Bari F (2017). Telekardiológia: A telemedicina feladatai és kötelességei [Telecardiology: Tasks and duties of telemedicine]. Orv Hetil.

[11] Noriega O, Ho H, Wright J (2014). The Application of Hand-Held Ultrasound Scanner in Teaching of Telemedicine and Rural Medicine. Donald School Journal of Ultrasound in Obstetrics and Gynecology.

[12] Bansal M, Singh S, Maheshwari P (2015). Value of interactive scanning for improving the outcome of new-learners in transcontinental tele-echocardiography (VISION-in-Tele-Echo) study. J Am Soc Echocardiogr.

[13] Ouzzani M, Hammady H, Fedorowicz Z, Elmagarmid A (2016). Rayyan-a web and mobile app for systematic reviews. Syst Rev.

[14] Buckley S, Coleman J, Davison I (2009). The educational effects of portfolios on undergraduate student learning: a Best Evidence Medical Education (BEME) systematic review. BEME Guide No. 11. Med Teach.

[15] Steinert Y, Mann K, Centeno A (2006). A systematic review of faculty development initiatives designed to improve teaching effectiveness in medical education: BEME Guide No. 8. Med Teach.

[16] Socransky S, Lang E, Bryce R, Betz M (2017). Point-of-Care Ultrasound for Jugular Venous Pressure Assessment: Live and Online Learning Compared. Cureus.

[17] Hempel D, Haunhorst S, Sinnathurai S (2016). Social media to supplement point-of-care ultrasound courses: the “sandwich e-learning” approach. A randomized trial. Crit Ultrasound J.

[18] Chenkin J, Lee S, Huynh T, Bandiera G (2008). Procedures can be learned on the Web: a randomized study of ultrasound-guided vascular access training. Acad Emerg Med.

[19] Cuca C, Scheiermann P, Hempel D, Via G, Seibel A, Barth M (2013). Assessment of a new e-learning system on thorax, trachea, and lung ultrasound. Emerg Med Int.

[20] Edrich T, Stopfkuchen-Evans M, Scheiermann P (2016). A Comparison of Web-Based with Traditional Classroom-Based Training of Lung Ultrasound for the Exclusion of Pneumothorax. Anesth Analg.

[21] Haskins SC, Feldman D, Fields KG (2018). Teaching a Point-of-Care Ultrasound Curriculum to Anesthesiology Trainees With Traditional Didactic Lectures or an Online E-Learning Platform: A Pilot Study. J Educ Perioper Med.

[22] Lian A, Rippey JCR, Carr PJ (2017). Teaching medical students ultrasound-guided vascular access - which learning method is best?. J Vasc Access.

[23] Maloney E, Hippe DS, Paladin A, Chew FS, Ha AS (2016). Musculoskeletal ultrasound training for radiology residents: lecture versus interactive learning module. Acad Radiol.

[24] Platz E, Liteplo A, Hurwitz S, Hwang J (2011). Are live instructors replaceable? Computer vs. classroom lectures for EFAST training. J Emerg Med.

[25] Platz E, Goldflam K, Mennicke M (2010). Comparison of Web-versus classroom-based basic ultrasonographic and EFAST training in 2 European hospitals. Ann Emerg Med.

[26] Bertran S, Boby H, Bertrand PM (2017). Comparison of video-based learning and lecture-based learning for training of ultrasound-guided central venous catheterization: a randomized controlled trial. BJA: British Journal of Anaesthesia.

[27] Soon AW, Toney AG, Stidham T, Kendall J, Roosevelt G (2020). Teaching Point-of-Care Lung Ultrasound to Novice Pediatric Learners: Web-Based E-Learning Versus Traditional Classroom Didactic. Pediatr Emerg Care.

[28] Brisson A-M, Steinmetz P, Oleskevich S, Lewis J, Reid A (2015). A comparison of telemedicine teaching to in-person teaching for the acquisition of an ultrasound skill - a pilot project. J Telemed Telecare.

[29] Fernández-Lao C, Cantarero-Villanueva I, Galiano-Castillo N, Caro-Morán E, Díaz-Rodríguez L, Arroyo-Morales M (2016). The effectiveness of a mobile application for the development of palpation and ultrasound imaging skills to supplement the traditional learning of physiotherapy students. BMC Med Educ.

[30] Cantarero-Villanueva I, Fernández-Lao C, Galiano-Castillo N (2012). Evaluation of e-learning as an adjunctive method for the acquisition of skills in bony landmark palpation and muscular ultrasound examination in the lumbopelvic region: a controlled study. J Manipulative Physiol Ther.

[31] Arroyo-Morales M, Cantarero-Villanueva I, Fernández-Lao C (2012). A blended learning approach to palpation and ultrasound imaging skills through supplementation of traditional classroom teaching with an e-learning package. Man Ther.

[32] Briz-Ponce L, Juanes-Méndez JA, García-Peñalvo FJ, García-Peñalvo FJ (2016). Proceedings of the Fourth International Conference on Technological Ecosystems for Enhancing Multiculturality - TEEM 16.

[33] Gul YA, Wan ACT, Darzi A (1999). Undergraduate surgical teaching utilizing telemedicine. Med Educ.

[34] Weber U, Zapletal B, Base E (2019). Resident performance in basic perioperative transesophageal echocardiography: Comparing 3 teaching methods in a randomized controlled trial. Medicine.

[35] Roubidoux MA, Chapman CM, Piontek ME (2002). Development and Evaluation of an Interactive Web-Based Breast Imaging Game for Medical Students. Acad Radiol.

[36] van Os MA, van der Ven AJ, Bloemendaal PM (2015). Effect of e-learning on quality of cervical-length measurements. Ultrasound Obstet Gynecol.

[37] Gotwald TF, Daniaux M, Stoeger A, Knapp R, zur Nedden D (2000). The value of the World Wide Web for tele-education in radiology. J Telemed Telecare.

[38] Piraino D, Recht M, Richmond B (1997). Implementation of an electronic teaching file using web technology. J Digit Imaging.

[39] Peters A, Patil PV (2019). Tele-echocardiography: enhancing quality at the point-of-care. Heart.

[40] Kim MS, Aro MR, Lage KJ (2016). Exploring the usability of mobile apps supporting radiologists’ training in diagnostic decision making. J Am Coll Radiol.

[41] Clavier T, Ramen J, Dureuil B (2019). Use of the Smartphone App WhatsApp as an E-Learning Method for Medical Residents: Multicenter Controlled Randomized Trial. JMIR Mhealth Uhealth.

[42] Ma M, Fallavollita P, Seelbach I (2016). Personalized augmented reality for anatomy education. Clin Anat.

[43] Marks A, Maizels M, Mickelson J (2011). Effectiveness of the computer enhanced visual learning method in teaching the society for fetal urology hydronephrosis grading system for urology trainees. J Pediatr Urol.

[44] Bretholz A, Doan Q, Cheng A, Lauder G (2012). A presurvey and postsurvey of a web- and simulation-based course of ultrasound-guided nerve blocks for pediatric emergency medicine. Pediatr Emerg Care.

[45] Long MC, Angtuaco T, Lowery C (2014). Ultrasound in telemedicine: its impact in high-risk obstetric health care delivery. Ultrasound Q.

[46] Rösch J (2001). Tele-education in Interventional Radiology. Cardiovasc Intervent Radiol.

[47] Robertson TE, Levine AR, Verceles AC (2017). Remote tele-mentored ultrasound for non-physician learners using FaceTime: A feasibility study in a low-income country. J Crit Care.

[48] Zimmermann R, Mousty E, Mares P, Letouzey V, Huberlant S (2019). E-learning et simulation en échographie focalisée pour la formation continue des sages-femmes en salle de naissance [Optimizing training in limited obstetric ultrasound for midwives through a combination of e-learning and simulation]. Gynecol Obstet Fertil Senol.

